# Immune cell early activation, apoptotic kinetic, and T-cell functional impairment in domestic pigs after ASFV CADC_HN09 strain infection

**DOI:** 10.3389/fmicb.2024.1328177

**Published:** 2024-02-14

**Authors:** Yunfei Tian, Dongyue Wang, Shicheng He, Zhen Cao, Wencai Li, Fei Jiang, Yifan Shi, Yuxin Hao, Xinhao Wei, Qingqing Wang, Shuai Qie, Jiangtao Wang, Ting Li, Xiaoli Hao, Jianzhong Zhu, Jiajun Wu, Shaobin Shang, Xinyan Zhai

**Affiliations:** ^1^College of Veterinary Medicine, Institute of Comparative Medicine, Yangzhou University, Yangzhou, China; ^2^The Biosafety High-Level Laboratory Management Office, China Animal Disease Control Center, Beijing, China; ^3^Animal Disease Control Center of Hunan Province, Changsha, China; ^4^Jiangsu Co-Innovation Center for Prevention and Control of Important Animal Infectious Diseases and Zoonosis, Yangzhou University, Yangzhou, China

**Keywords:** ASFV, apoptotic, T cell early activation, cytokine, immunopathogenesis

## Abstract

African swine fever (ASF) caused by the African swine fever virus (ASFV) is a fatal and highly contagious disease of domestic pigs characterized by rapid disease progression and death within 2 weeks. How the immune cells respond to acute ASFV infection and contribute to the immunopathogenesis of ASFV has not been completely understood. In this study, we examined the activation, apoptosis, and functional changes of distinct immune cells in domestic pigs following acute infection with the ASFV CADC_HN09 strain using multicolor flow cytometry. We found that ASFV infection induced broad apoptosis of DCs, monocytes, neutrophils, and lymphocytes in the peripheral blood of pigs over time. The expression of MHC class II molecule (SLA-DR/DQ) on monocytes and conventional DCs as well as CD21 expression on B cells were downregulated after ASFV infection, implying a potential impairment of antigen presentation and humoral response. Further examination of CD69 and *ex vivo* expression of IFN-γ on immune cells showed that T cells were transiently activated and expressed IFN-γ as early as 5 days post-infection. However, the capability of T cells to produce cytokines was significantly impaired in the infected pigs when stimulated with mitogen. These results suggest that the adaptive cellular immunity to ASFV might be initiated but later overridden by ASFV-induced immunosuppression. Our study clarified the cell types that were affected by ASFV infection and contributed to lymphopenia, improving our understanding of the immunopathogenesis of ASFV.

## Introduction

African swine fever (ASF) caused by the African swine fever virus (ASFV) is a fatal and highly contagious disease of domestic pigs and wild boars, with a mortality rate of up to 100% (Galindo and Alonso, 2017). Since its first identification in Kenya in 1921 (Cwynar et al., 2019), this disease has spread from Africa to Europe and Asia, affecting many countries. In August 2018, the first case of ASF was documented in Shenyang, China, and since then, ASF has caused tremendous economic losses and has been the most devastating disease affecting the swine industry in China (Ge et al., 2018; Zhou et al., 2018; Zhao et al., 2019). ASFV is a double-stranded DNA virus and the only member of the Asfarviridae family. The ASFV genome has 170–190 kb nucleotides and contains 151–167 open reading frames (ORFs), depending on the virus strain (Dixon et al., 2013), which encode 54 structural proteins and more than 100 non-structural proteins (Alejo et al., 2018) involved in viral replication and assembly as well as in modulating host cellular functions and immune evasion (Galindo and Alonso, 2017).

Virulent ASFV infection generally leads to an acute/peracute hemorrhagic disease in domestic pigs, and the infected pigs usually die within 2 weeks (6–9 days for experimental inoculation, and 13–14 days post-contact) (Zhao et al., 2019). However, the detailed mechanism underlying rapid disease progression and death has not been completely understood (Takamatsu et al., 2013; Pikalo et al., 2019). It was known that ASFV mainly infects myeloid lineage cells, including monocytes, neutrophils, macrophages (Carrasco et al., 1996a; Gomez-Villamandos et al., 2013), and dendritic cells (DCs) (Franzoni et al., 2018). Severe leucopenia or lymphopenia was evident in the periphery blood and lymphoid organs of ASFV-infected pigs due to apoptosis (Oura et al., 1998; Karalyan et al., 2012), accompanied by the emergence of immature cells and atypical lymphocytes (Karalyan et al., 2012). However, the cell types that were affected by ASFV infection and contributed to lymphopenia have not been well defined, though *in situ* apoptotic immune cells were primarily examined by the terminal deoxynucleotidyl transferase (TdT) dUTP Nick-end labeling (TUNEL) technique (Oura et al., 1998).

In addition, ASFV infection also results in an exacerbated inflammatory cytokine storm, which is characterized by sustained elevation of serum pro-inflammatory interleukins, tumor necrosis factor (TNF), and chemokines (Wang et al., 2020; Franzoni et al., 2023), and TNF-α is required for cell apoptosis in porcine alveolar macrophages (PAMs) after ASFV infection (Zheng et al., 2022). There was no direct evidence showing that T cells were activated or IFN-γ was released by specific cell types, though the transient increase of IFN-γ was observed from day 4–6 post-inoculation (Karalyan et al., 2012). Recent studies showed that an infection of the highly virulent ASFV strain led to the reduction of distinct T-cell subsets to some degree, except for CD4^+^CD8^+^ T cells in domestic pigs (Huhr et al., 2020), whereas the moderate virulent ASFV strain induced an increase in CD8α^+^ and CD4^+^CD8α^+^ αβ T cells and delayed the proliferation of CD8α^+^ T cells in domestic pigs (Schafer et al., 2021). However, whether T cells are activated or functionally impaired during rapid disease progression and death caused by virulent ASFV infection is still lacking in evidence. The dynamics of distinct immune cell activation and apoptosis as well as functional changes of T cells during ASFV infection remain to be clarified, in order to better understand the immunopathogenesis of ASFV.

In this study, we examined the activation, apoptosis, and functional changes of distinct immune cells in domestic pigs following acute infection with the ASFV CADC_HN09 strain using multicolor flow cytometry, in combination with an anti-pig CD69 antibody we developed previously (Tian et al., 2022) and *ex vivo* staining of IFN-γ-producing cells. Our results showed that there was transient T-cell activation and functional impairment as well as broad apoptosis on distinct immune cells after virulent ASFV infection.

## Materials and methods

### Ethics statement

All experiments involving ASFV were approved by the Institutional Biosafety Committee of the Ministry of Agriculture and Rural Affairs of China (07140020210615–1) and performed in animal biosafety level 3 (ABSL-3) facilities, in accordance with the institutional biosafety manual of China Animal Disease Control Center. All the protocols for animal studies complied with the guidelines of the Animal Welfare and Ethics of China Animal Disease Control Center.

### Animals and virus

Piglets for the ASFV infection experiment were purchased from Beijing Qingquanwan Pig Breeding Co. LTD, were unvaccinated with vaccines, and were routinely pre-detected for ASFV, PRRSV, PRV, PCV, and PEDV by RT-qPCR. The ASFV CADC_HN09 strain was isolated and sequenced (GenBank accession number: MZ614662) in 2021, belongs to genotype II, and is kept in China Animal Disease Control Center.

### Infection experiments with ASFV

In experiment 1, eight 2-month-old ASFV-free healthy piglets were randomly divided into two groups. One group (five pigs) was intramuscularly infected with 10 hemadsorbing doses (HAD_50_) of ASFV CADC_HN09 strain per piglet while a control group (three pigs) was uninfected (see Table 1). Periphery blood of each pig was collected at 0, 3, 5, and 7 days post-infection (dpi) for the isolation of PBMCs and immunophenotyping by flow cytometry (FCM).

**Table 1:** Infection experiments and sample collection.

Days post-infection		0	3	5	7
Experiment 1 (blood)	Uninfected	3	3	3	3
	Infected	5	5	5	3
Experiment 2 (organs)	Uninfected		3	3	3
	Infected		3	3	3

In experiment 2, 18 2-month-old healthy piglets were randomly divided into two groups (nine pigs in each group). One group was intramuscularly infected with 10 HAD_50_ of ASFV CADC_HN09 strain per piglet, and the other group was left uninfected and served as control at 3, 5, and 7 dpi. Pigs were euthanized at each time-point, and the lung, spleen, and mandibular lymph node (mLN) were collected for single-cell suspension, serum preparation, and immunophenotyping by flow cytometry.

Clinical signs and rectal temperatures of the piglets were recorded daily over the course of the experiment. The clinical score was evaluated as previously described (Petrov et al., 2018), which comprised the parameters of liveliness, bearing, breathing, gait, skin, and feed uptake.

### Single-cell preparation

Single-cell suspension from peripheral blood and organs was prepared according to a previous study (Hao et al., 2020; Tian et al., 2022). Briefly, PBMCs were isolated from the heparinized periphery blood of pigs by gradient density centrifugation with a Porcine PBMC Isolation Kit (Tianjin Haoyang Co. Ltd., China). Single-cell suspensions from mLN and spleen were prepared by grinding the tissues through 70-μm cell strainers (BD, United States) in 2% fetal bovine serum (FBS) RPMI-1640 medium. Lung samples were predigested with collagenase IV and DNase I (Sigma, Germany) for 30 min at 37°C in a water bath and then ground, followed by isolation with a Porcine Lymphocyte Isolation Kit (Tianjin Haoyang Co. Ltd., China). Red blood cells in samples were lysed with red blood cell lysis buffer (Solarbio, China). Live cells were counted using a hemocytometer, and the cell concentration was adjusted to 2 × 10^7^ cells/mL.

### Flow cytometry

Single-cell suspensions from blood and organs were seeded in 96-well V-bottom plates, with 2 × 10^6^ cells in each well. After centrifugation, cells were incubated for 30 min at RT in the dark with a cocktail of fluorescence-conjugated antibodies or biotinylated antibodies and/or followed by a secondary antibody staining, as previously reported (Maisonnasse et al., 2016; Lagumdzic et al., 2023). Antibodies used in the FCM are summarized in Table 2. The early apoptosis of myeloid lineage cells and lymphocytes was detected using an Annexin V detection kit (Biolegend, United States), cytokine secretion of lymphocytes was detected by intracellular cytokine staining (ICS), and the proliferation of lymphocytes was examined by intranuclear transcriptional factor staining, with different staining panels. After each staining, a washing step was performed with FCM buffer (0.5% BSA PBS) by centrifuging at 400 g for 5 min at 4°C. A minimal number of 300,000 cells was acquired for FCM analysis. Live singlets in PBMCs were gated based on their forward scatter (FSC) and side scatter (SSC) properties, together with a negative fixable viability dye eFluor® 780 (FVD780, Thermo Scientific, United States) signal. FCM was performed with FACS LSRFortessa (BD Biosciences, Franklin Lakes, NJ, United States), and the data were analyzed by FlowJo software v10.6.2 (Tree Star Inc., Ashland, OR, United States).

**Table 2:** Antibodies used in this study.

Antigen	Clone	Isotype	Fluorochrome	Source
CD3ε	BB23-8E6-8C8	Mouse IgG2a, κ	PerCP-Cy™5.5	BD
CD4a	74–12-4	Mouse IgG2b, κ	PE-Cy™7	BD Pharmingen
CD8a	76–2-11	Mouse IgG2a, κ	Biotin	Southernbiotech
CD21	BB6-11C9.6	Mouse IgG1, κ	AlexaFluor®488	Southernbiotech
γδTCR	MAC320	Rat PVG IgG2a	PE	BD
CD69	5F12	Mouse IgG1, κ	Dylight755	in-house
CD163	2A10/11	Mouse IgG1	RPE	Bio-rad
CD172a	74–22-15	Mouse IgG1, κ	FITC	Southernbiotech
SLA II DR	2E9/13	IgG2b	APC	Bio-rad
SLA II DQ	K274.3G8	IgG1	APC	Bio-rad
IL-2	A150D3F1	Mouse IgG2a	FITC	Invitrogen
IFN-γ	P2G10	Mouse IgG1, κ	Alexa Fluor® 647	BD
TNF-α	MAB11	Mouse IgG1, κ	BV 421™	BioLegend
Ki67	B56	Mouse IgG1, κ	BV 421™	BD Horizon™
Foxp3	FJK-16 s	Rat IgG2a, κ	PE	Invitrogen
Biotin	–		BV 510™	BioLegend

### Apoptosis detection

PBMCs were seeded in 96-well V-bottom plates, with 2 × 10^6^ cells in each well and stained first with FVD780 in pre-cold PBS for 10 min at 4°C. Then, the cells were stained with antibody cocktails containing fluorescein-conjugated antibodies to CD172, CD163, SLA-II DR/DQ, or CD21, γδTCR, CD3, CD4, and CD8α, followed by a second antibody staining in FCM buffer at RT for 30 min. Thereafter, cells were washed with FCM buffer, and stained with fluorescein-labeled Annexin V diluted in Annexin V binding buffer (Biolegend, United States) for 15 min at 4°C. Cells were washed and resuspended with Annexin V binding buffer and immediately analyzed by FCM.

### Intracellular cytokine staining

For *in vitro* activation of lymphocyte, 2 × 10^6^ PBMCs per sample were seeded in 96-well U-bottom plates kept in a 200-μL RPMI-1640 complete medium containing 10% FBS, 100 U/mL penicillin, and 100 mg/mL streptomycin (Gibco, United Kingdom) and stimulated for 6 h with 50 ng/mL PMA and 500 ng/mL ionomycin in the presence of 10 μg/mL Brefeldin A (Biolegend, United States).

After incubation, cells were stained for cell surface markers for 30 min at RT, washed with FCM buffer, and fixed with 4% paraformaldehyde for 10 min; then, the cells were permeabilized with Perm/wash buffer (BD, United States) for 20 min, and stained with Alexa Fluor® 647 Mouse Anti-Pig IFN-γ (BD, United States), Brilliant Violet 421™ anti-human TNF-α (BioLegend), and FITC-conjugated anti-IL-2 mAbs (Thermo Fisher, United States) diluted in Perm/wash buffer for 30 min in dark at RT. After washing with Perm/wash buffer, the cells were resuspended with 0.5% PFA PBS buffer and examined by FCM. For *ex vivo* detection of IFN-γ production by T cells, freshly isolated PBMCs were directly stained with cell surface markers and then subjected to the intracellular staining protocol.

### Intranuclear transcriptional factor staining

Intranuclear Ki67 and Foxp3 factors were stained as below. Briefly, cells were first stained with antibodies against surface markers CD21, γδTCR, CD3, CD4, and CD8α. Then, the cells were fixed and permeabilized with fixation/permeabilization buffer (Invitrogen, United States) for 45 min and stained with mouse anti-Ki67 (BD Horizon™, United States) and/or anti-Foxp3 (Invitrogen, United States) diluted in permeabilization buffer (Invitrogen, United States) for another 45 min. After final washing with the permeabilization buffer, cells were resuspended with 0.5% PFA PBS buffer and examined by FCM.

### ELISA

Pig sera were isolated at 5 dpi for the detection of IL-1β and IL-18 using a commercial ELISA kit (Thermo Fisher, United States). The test was carried out according to the manufacturer’s protocol.

### Statistical analysis

Statistical analysis was performed with GraphPad Prism software (GraphPad, La Jolla, CA). When comparing experimental values from two groups, Student’s t-tests were routinely used. Statistical significance is noted (*p < 0.05; **p < 0.01; ***p < 0.001).

## Results

### Clinical manifestations

After inoculation of ASFV CADC_HN09, the piglets showed a loss of appetite, depression, lethargy, skin cyanosis, and dyspnea. Most of the infected piglets showed high fever (over 41°C) that started from 5 dpi and lasted until the end (Supplementary Figure S1), consistent with a previous report (Zhao et al., 2019). Two piglets were euthanized at 6 dpi due to severe clinical signs.

### Virulent ASFV infection led to the predominant reduction of B cells, CD4 T cells, monocytes, and dendritic cells in periphery blood

**Figure 1:**
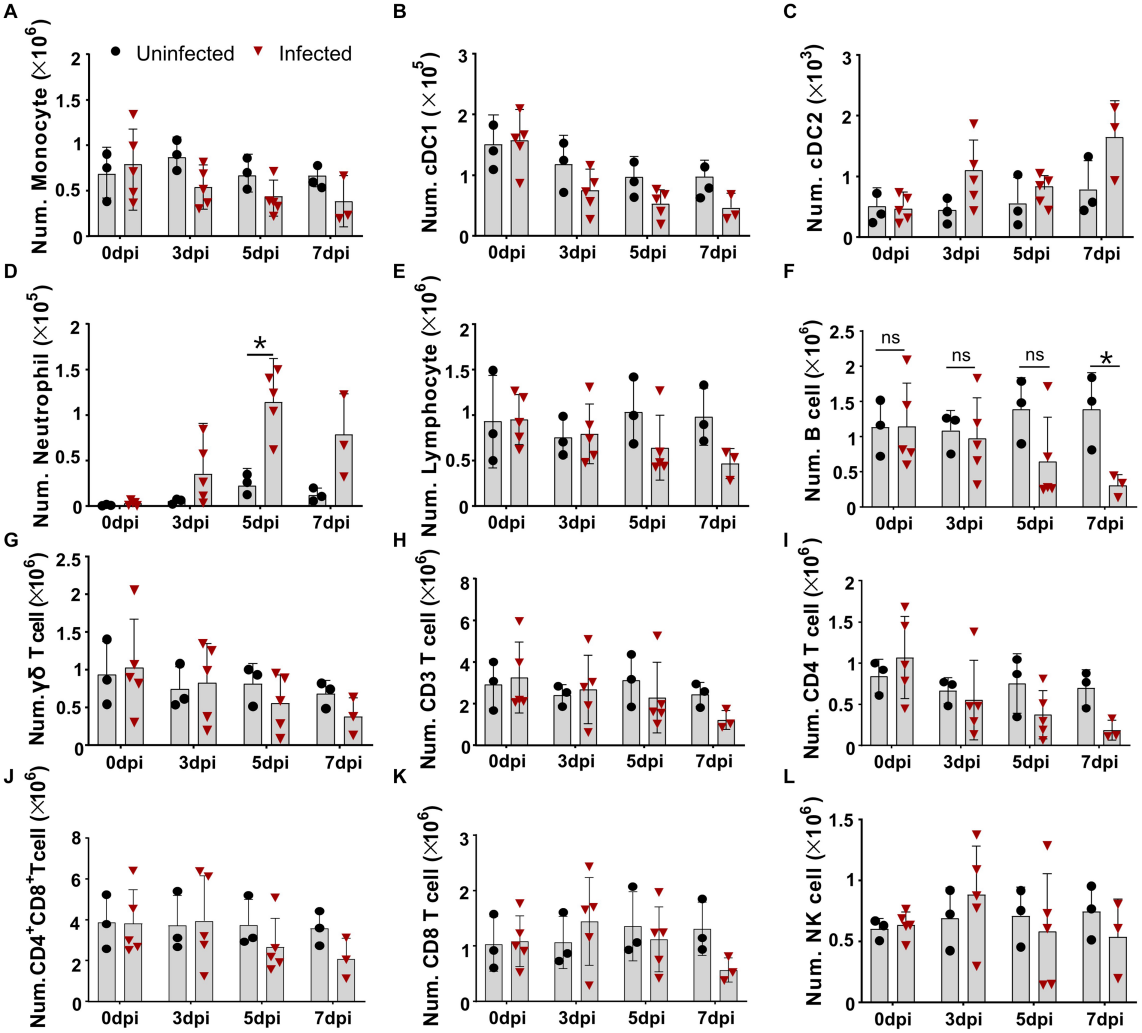
Virulent ASFV infection led to predominant reduction of B cells, CD4 T cells, monocytes, and DCs in periphery blood. At the indicated time points, PBMCs were prepared and stained for distinct leukocyte subsets and were analyzed by FCM. The absolute amount of each leukocyte subset per milliliter of blood was calculated based on the percentage of the specific cell type in live PBMCs. The numbers of monocyte **(A)**, cDC1 **(B)**, cDC2 **(C)**, neutrophil **(D)**, lymphocyte **(E)**, B cell **(F)**, γδ T cell **(G)**, CD3 T cell **(H)**, CD4 T cell **(I)**, CD4^+^CD8^+^ T cell **(J)**, CD8 T cell **(K)**, and NK cell **(L)** in 1 mL blood. Data shown are mean ± SD. *p < 0.05. **p < 0.01.

As lymphopenia was previously evident in the tissues of the infected pigs, but the cell types were not completely identified, we intended to identify the changes of distinct immune cells in the periphery blood of ASFV-infected pigs. We first defined different immune cell subsets from myeloid lineage by multicolor flow cytometry according to a previous report (Maisonnasse et al., 2016). As shown in Figure 1 and Supplementary Figure S2A, in terms of numbers, MHC II^+^CD172a^+^CD163^+^ monocytes and MHC II^+^CD172a^−^CD163^−^ conventional dendritic cells type I (cDC1) gradually decreased over the course of infection (Figures 1A,B), while MHC II^+^CD172a^+^CD163^−^ conventional dendritic cell type II (cDC2) and neutrophils (MHC II^−^CD163^−^CD172^+^) significantly increased after infection (Figures 1C,D). As the PBMC isolation kit we used could not completely remove granulocytes, neutrophil was also analyzed in this study. Further analyzing the changes in lymphocyte subsets showed that total lymphocytes, total T cells (CD3^+^), and γδ T cells slowly decreased (Figures 1E–G; Supplementary Figure S2B) over the course of the disease, while CD21^+^ B cells and CD4^+^ T cells (CD3^+^CD8^−^CD4^+^) decreased more drastically from 5 dpi in terms of numbers (Figures 1H,I). The numbers of CD4^+^CD8^+^ T cells and CD8^+^ T cells were relatively stable until 7 dpi, after which they decreased, whereas the number of NK cells (CD3^−^CD8^+^ T) showed a slight increase and then decreased (Figures 1J–L). In contrast, the corresponding cell populations in the uninfected pigs were not much changed, suggesting that technical and experimental variations at each timepoint were minimized.

### Virulent ASFV infection caused different levels of apoptosis on distinct immune cells

**Figure 2:**
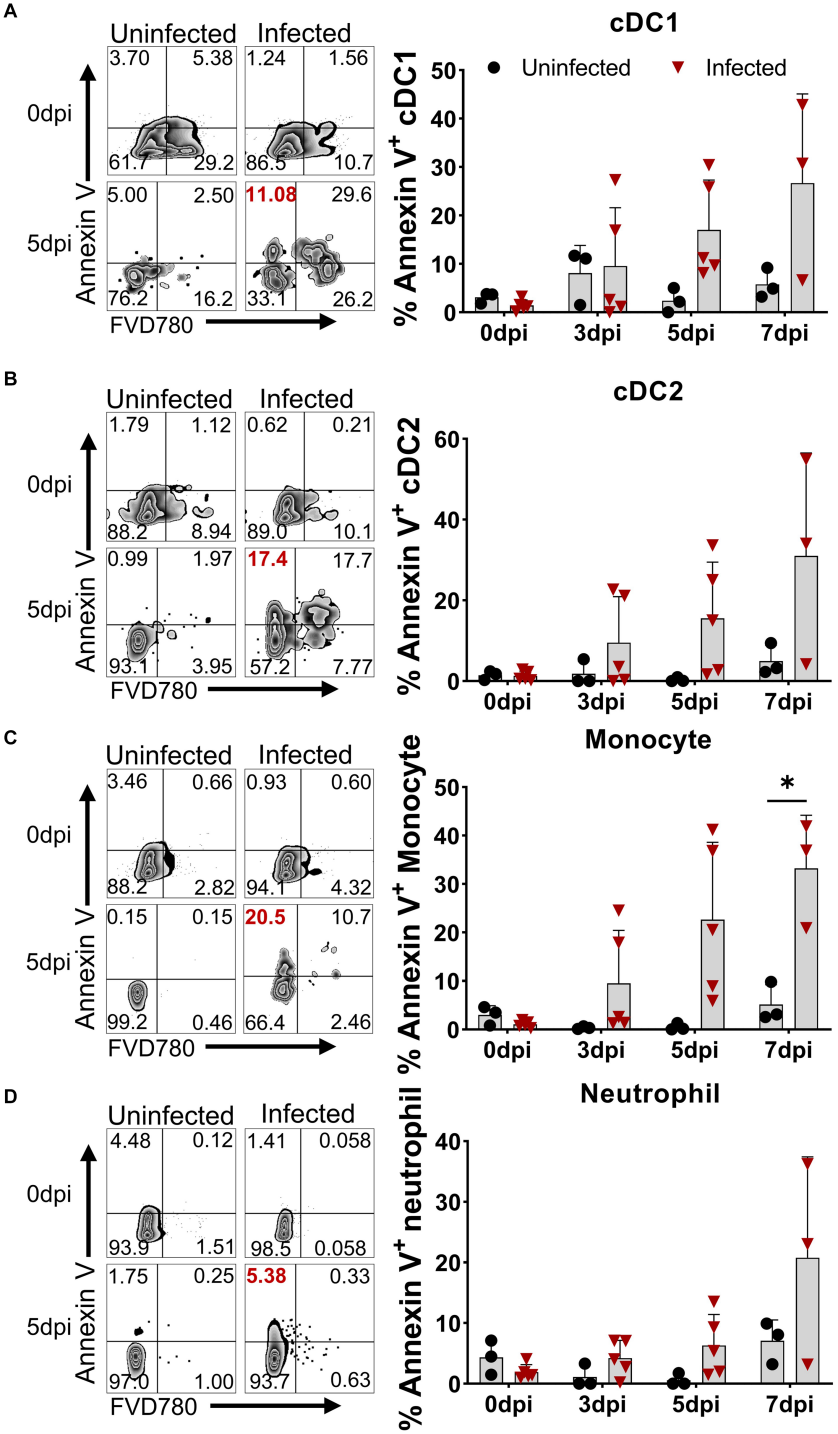
Apoptosis of DCs, monocytes, and neutrophils *in vivo* after ASFV infection. At the indicated time points, PBMCs of ASFV-infected and ASFV-uninfected piglets were prepared and stained for the apoptosis of cDC1, cDC2, monocytes, and neutrophils and were analyzed by FCM. Representative zebra plots (left panel) and kinetic changes (right panel) of the percentage of apoptotic (Annexin V^+^) cDC1 **(A)**, cDC2 **(B)**, monocytes **(C)**, and neutrophils **(D)**. FVD780^+^ cells, dead cells. Data shown are mean ± SD. *p < 0.05.

Although apoptosis was detected in porcine macrophage during *in vitro* infection with high and low virulent ASFV isolates (Ramiro-Ibanez et al., 1996; Portugal et al., 2009) and in lymphocytes in the liver, kidney tissues, and lymphoid organs of the infected pigs (Gomez-Villamandos et al., 1995; Ramiro-Ibanez et al., 1996; Carrasco et al., 1996b), *in vivo* apoptotic kinetics of distinct immune cells in PBMCs after virulent ASFV infection have not been examined. We first examined the expression of Annexin V on the cell surface for measuring the early apoptosis of myeloid lineage subsets including DCs, monocytes, and neutrophils using multicolor flow cytometry. The results showed that early apoptosis (Annexin V-positive cells) occurred as early as 3 dpi in DCs, monocytes, and neutrophils in PBMCs to various extents (Figure 2). The kinetics of apoptosis in these cells peaked at 7 dpi, in which the percentages of apoptotic cDC1 (Figure 2A), cDC2 (Figure 2B), monocytes (Figure 2C), and neutrophils (Figure 2D) reached an average of 26.71 ± 18.42%, 31.03 ± 25.49%, 33.23 ± 10.97%, and 20.78 ± 16.64%, respectively.

**Figure 3:**
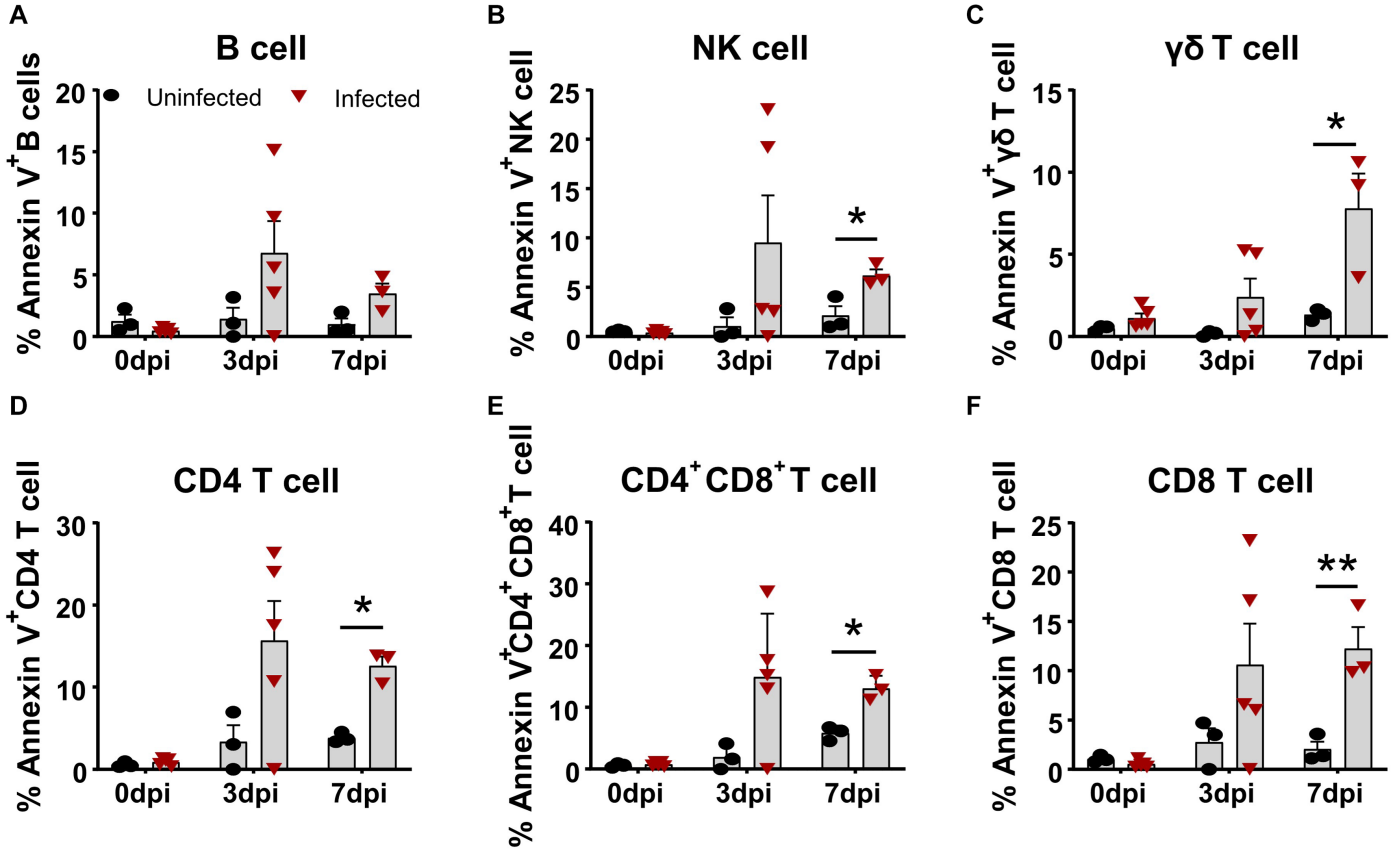
Apoptosis of T cell subsets *in vivo* after ASFV infection. At the indicated time points, PBMCs of ASFV-infected and ASFV-uninfected piglets were prepared and stained for apoptotic T cell subsets and were analyzed by FCM. Kinetic changes of the percentage of apoptotic (Annexin V^+^) B cell **(A)**, NK cell **(B)**, γδ T cell **(C)**, CD4 T cell **(D)**, CD4^+^CD8^+^ T cell **(E)**, and CD8^+^ T cell **(F)**. FVD780^+^ cells, dead cells. Data shown are mean ± SD. *p < 0.05; **p < 0.01.

Similarly, we simultaneously examined the apoptosis of several subsets of lymphocytes in terms of Annexin V expression. As shown in Figure 3, all lymphocyte subsets of the infected animals showed different levels of apoptosis from 3 dpi on, compared to the control. The frequency of apoptotic B cells and NK cells increased to 6.78 ± 2.60% and 9.51 ± 4.79% (Figures 3A,B) at 3 dpi, respectively. The percentage of apoptotic γδ T cells gradually increased from 2.39 ± 2.55% at 3 dpi to 7.79 ± 3.71% at 7 dpi (Figure 3C), whereas αβ T cells showed higher proportional Annexin V-positive cells. Among these T cells, the frequencies of apoptotic CD4^+^ T cells and CD4^+^CD8^+^ T cells peaked at 3 dpi, ranging from 10 to 30% with an average of 15% (Figures 3D,E), whereas Annexin V^+^CD8^+^ T cells showed relatively lower proportion, with an average of 10.59 ± 4.18% (Figure 3F) that sustained until 7 dpi.

Considering that acute virulent ASFV infection may cause more than one type of cell death, we detected the levels of IL-1β and IL-18, which are products of pyroptosis (He et al., 2015, Gao et al., 2022) in pig sera at 5 dpi. As shown in Supplementary Figure S3, the levels of IL-1β and IL-18 in infected pig sera increased compared with that of uninfected pig sera.

### Virulent ASFV infection downregulated the expression of MHC II and CD21 molecules on APCs and B cells, respectively

**Figure 4:**
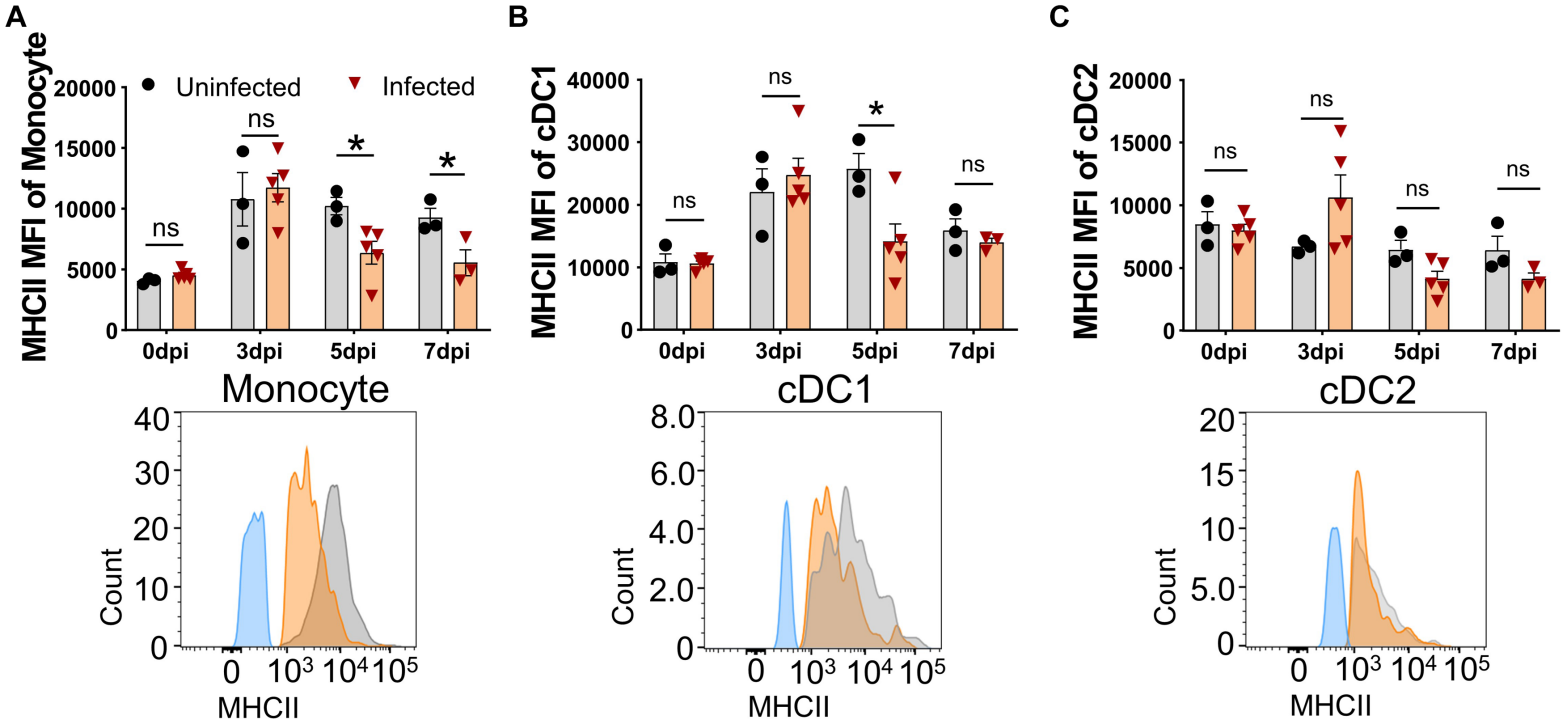
Downregulation of MHC II on APCs after ASFV infection. At the indicated time points, PBMCs of piglets infected with or without ASFV were prepared and stained for monocyte and DCs and were analyzed by FCM. MFI of MHC II on monocyte **(A)**, cDC1 **(B)**, and cDC2 **(C)** was calculated for assessment of the expression level on each subset (upper panel). Each point represents data from a single piglet, while bars represent the mean of each group. A representative histogram of the MHC II^+^ APCs at 5 dpi (lower panel); the black line represents uninfected piglets and the orange line represents infected piglets, while the blue line means MHC II-FMO control. Data shown are mean ± SD. ns, no statistical significance. *p < 0.05; **p < 0.01.

The upregulation of the MHC II molecule on the cell surface is a hallmark of antigen-presenting cell (APC) maturation, especially DC maturation (Franzoni et al., 2019), and is associated with the capability of DCs to present antigen and prime T cells (Dalod et al., 2014). It was unclear whether ASFV infection affected the MHC II expression on APCs *in vivo*. Therefore, we examined the expression of SLA-DR/DQ molecules and their mean fluorescence intensity (MFI) on APCs by FCM. As shown in Figure 4, the MFI of SLA-DR/DQ on monocytes and cDC1 from infected piglets significantly decreased from 5 dpi on (Figures 4A,B), while the MFI of MHC II on cCD2 did not show any obvious change (Figure 4C). These results indicated that infection with ASFV CADC_HN09 strain may affect the antigen-presenting function of porcine monocytes and cDC1.

**Figure 5:**
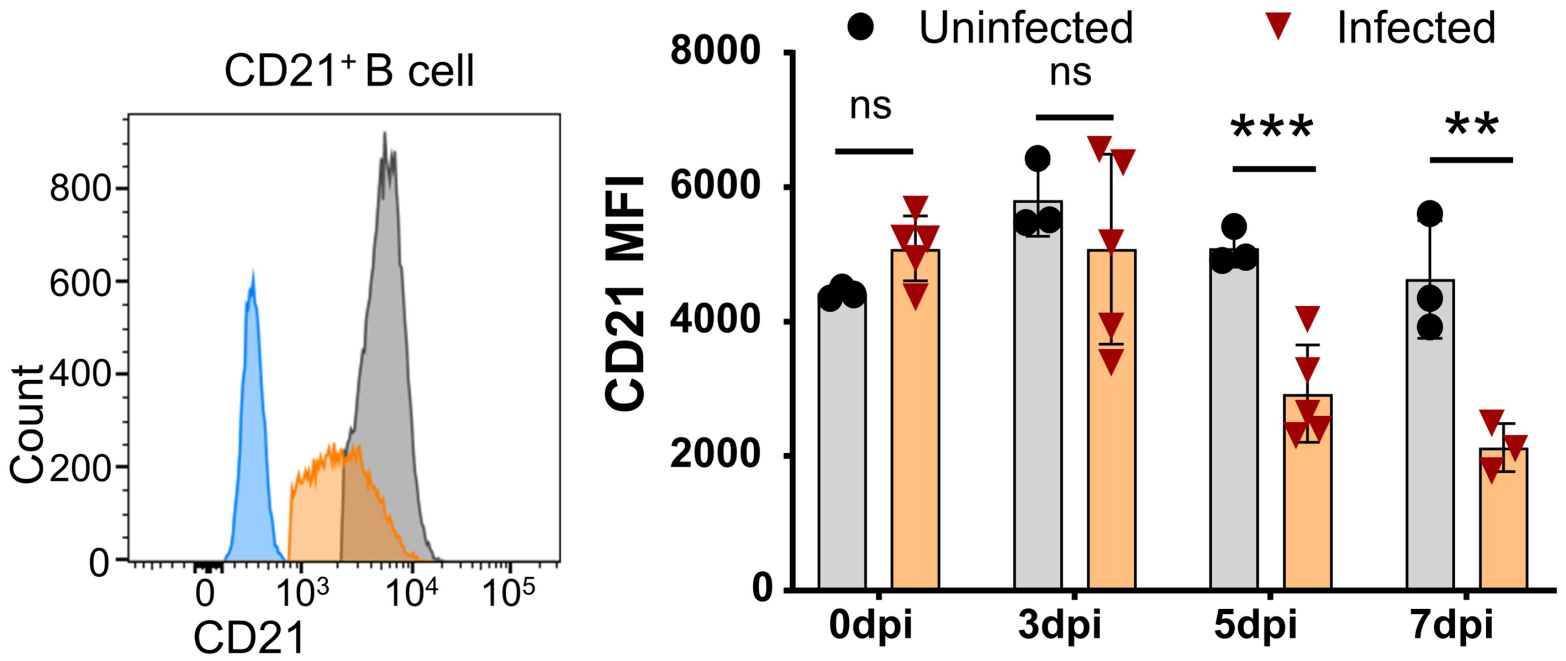
Downregulation of CD21 on B cells after ASFV infection. At the indicated time points, PBMCs of ASFV-infected or ASFV-uninfected piglets were separated and stained for FCM analysis of B cells. A representative histogram of CD21^+^ B cell at 5 dpi (left panel), and the black line represents uninfected piglets and the orange line represents infected piglets, while the closed blue histogram means FITC-CD21-FMO control. Dynamic changes of CD21 MFI on B cells (right panel). Each point represents data from a single pig, while the bars represent the mean of each group. Data shown are mean ± SD. ns, no statistical significance. **p < 0.01; ***p < 0.001.

Further analysis showed that CD21 MFI was significantly decreased as early as 5 dpi and further decreased at 7 dpi (Figure 5), while the percentage of CD21^+^ B cells was reduced during ASFV infection. Given that CD21 is a marker for the maturation and activation of naive B cells and is associated with complement activation (Ahearn and Fearon, 1989), this result suggested that ASFV infection may potentially impair B cell development and complement activation.

### African swine fever virus infection induced transient activation of T cells and NK cells

**Figure 6:**
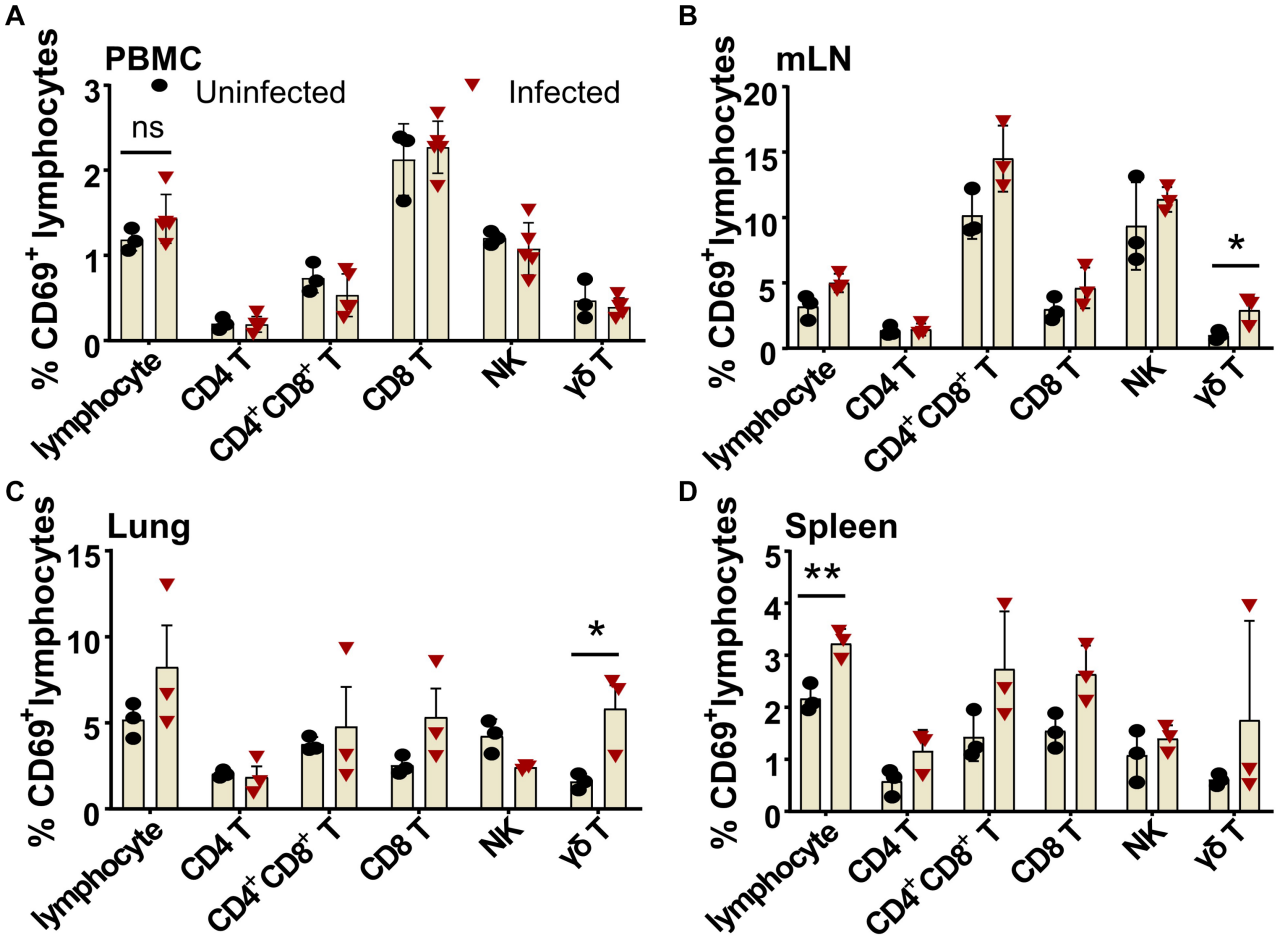
Early activation of different lymphocyte subsets after ASFV infection in blood and organs. Single-cell suspensions of peripheral blood, mLN, lung, and spleen were prepared at 3 dpi and stained for lymphocyte subsets and were analyzed by FCM. The percentages of CD69^+^ lymphocyte subsets in PBMC **(A)**, mLN **(B)**, lung **(C)**, and spleen **(D)** were compared between ASFV-uninfected and ASFV-infected groups. Data shown are mean ± SD. *p < 0.05.

The ASF disease progression was so rapid that the infected pigs usually died within 7–14 days after infection (Zhao et al., 2019). It was debatable whether there is an activation of lymphocytes after virulent ASFV infection. Using porcine CD69 as the very early activation marker (Tian et al., 2022) and *ex vivo* cytokine staining, we examined the early activation of lymphocytes after ASFV infection. As shown in Figure 6, as early as 3 dpi, though no obvious early activation of lymphocytes was detected in PBMCs (Figure 6A), the percentages of CD69^+^ γδ T cells in both mLN (2.91 ± 0.63% vs. 1.01 ± 0.20%) and lungs (5.8 ± 1.38% vs. 1.57 ± 0.27%) were significantly increased in the ASFV-infected pigs, compared to the uninfected pigs (Figures 6B,C). Interestingly, the early activation of lymphocytes (3.22 ± 0.16% vs. 2.17 ± 0.15%) appeared first in the spleen of the infected pigs and was more significant than in other organs (Figure 6D). Among the activated lymphocytes, distinct T-cell subsets (CD4^+^, CD4^+^CD8^+^, and CD8^+^ T cells) showed obviously higher CD69 expression than those cells from the control. The activation of these cells became even more evident at 5 dpi and more pronounced in lymphoid organs than in periphery blood in terms of CD69 expression after infection, which was shown in our previous study (Tian et al., 2022).

**Figure 7:**
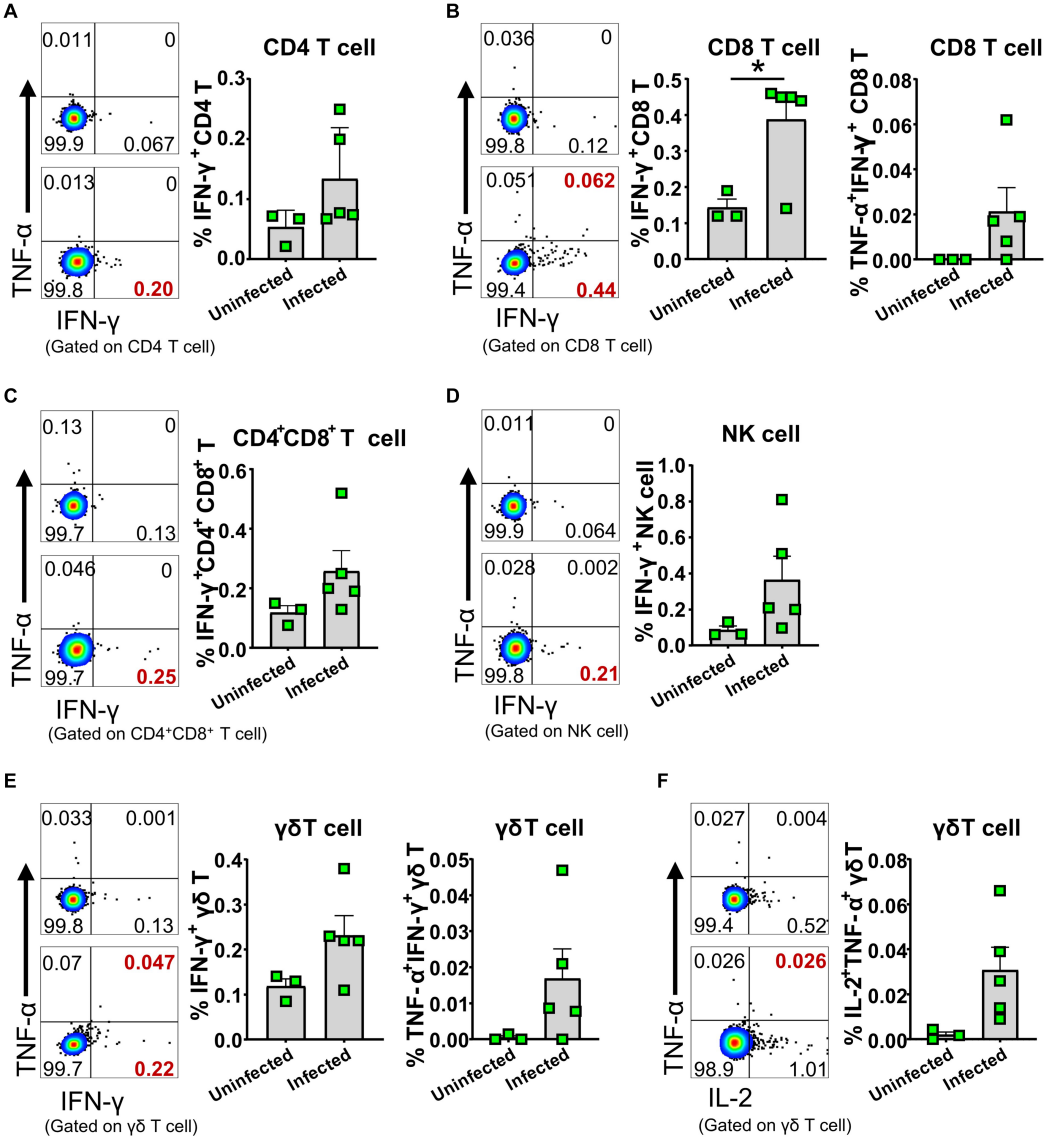
ASFV infection induced cytokine-secreting T cells and NK cells. PBMC of both ASFV-infected and ASFV-uninfected piglets were separated at 5 dpi and stained for lymphocyte subsets, followed by intracellular cytokine staining for TNF-α, IFN-γ, and IL-2, and then, the cells were analyzed by FCM. Representative pseudocolor plots (left panel: the upper one, uninfected; the lower one, infected) and increased frequencies of IFN-γ^+^, IFN-γ^+^TNF-α^+^ or IL-2^+^ CD4 T **(A)**, CD8 T **(B)**, CD4^+^CD8^+^ T **(C)**, NK **(D)** and γδ T cells **(E,F)** (right panel). Data shown are mean ± SD. *p < 0.05.

To further identify whether the activated lymphocytes express cytokines *in vivo* upon ASFV infection, we performed an *ex vivo* intracellular cytokine staining of freshly isolated PBMCs at 5 dpi. We found that there were higher percentages of IFN-γ-secreting CD4^+^, CD8^+^ and CD4^+^CD8^+^ T cells, γδ T cells, and NK cells (Figures 7A–E; Supplementary Figure S2F), as well as TNF-α and IL-2-secreting γδ T cells (Figure 7F) in the ASFV-infected pigs, compared to the uninfected pigs, though the percentages were relatively low. In addition, more frequencies of TNF-α^+^IFN-γ^+^ CD8^+^ T cells and γδ T cells were observed in the ASFV-infected pigs (Figures 7B,E). These results indicated that there was *in vivo* activation of T cells and NK cells during virulent ASFV infection.

### African swine fever virus infection impaired the capability of αβ T cells to produce cytokines

**Figure 8:**
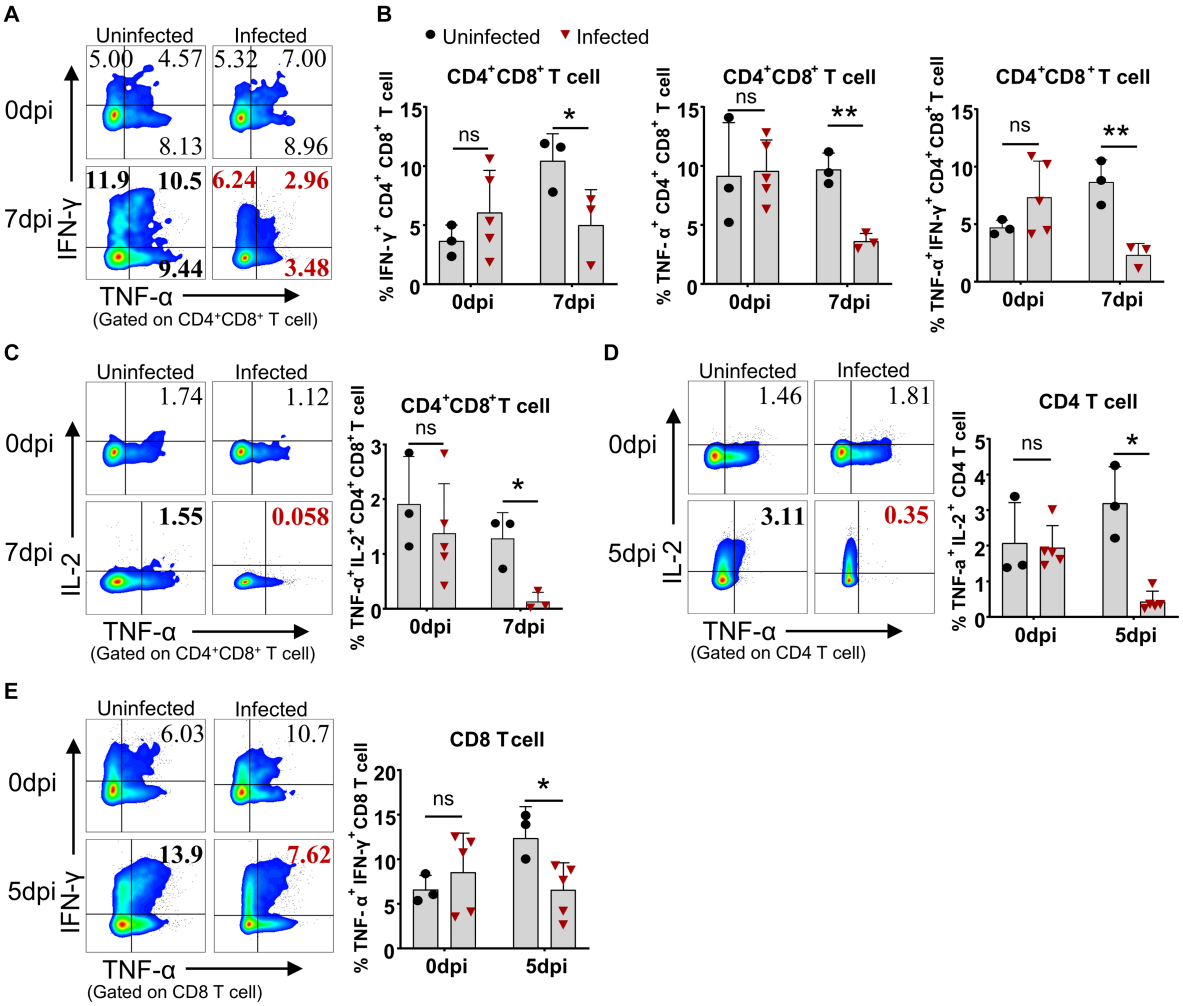
ASFV infection impaired the capability of T cells to produce cytokines. At the indicated time points, PBMC of ASFV-infected and uninfected piglets were separated and stimulated with mitogen and were stained for lymphocyte subsets, followed by an intracellular cytokine staining step and cells were determined by FCM. Representative pseudocolor plots **(A)** and the differential expression of cytokines in CD4^+^CD8^+^ T cell **(B)**. Representative pseudocolor plots (left panels) and the differential expression of cytokines in CD4^+^CD8^+^ T cell **(C)**, CD4 T cell **(D)** and CD8 T cell **(E)** (right panels). Each point represents data from a single pig while bars represent the mean of each group. Data shown are mean ± SD. ns, no statistical significance. *p < 0.05; **p < 0.01.

Even though the lymphocytes of the infected pigs were activated, these animals died in 7 to 9 days after infection in this study, suggesting the adaptive immunity to ASFV may be overridden by the immunosuppression caused by ASFV. Therefore, we compared the capability of lymphocytes from the infected and uninfected pigs to produce cytokines upon mitogenic stimulation by flow cytometry. The results showed that CD4^+^CD8^+^ T cells from the infected piglets were less capable of producing IFN-γ (5.0 ± 3.0% vs 10.43 ± 2.3%) and TNF-α (3.61 ± 0.68% vs 9.72 ± 1.36%) at 7 dpi upon PMA/Ionomycin stimulation, compared to those from the uninfected pigs (Figures 8A,B). Moreover, the results also revealed that CD4^+^CD8^+^ T and CD4 T cells showed reduced polyfunctionality for producing both TNF-α and IL-2 (0.13 ± 0.16% vs 1.28 ± 0.48% and 0.44 ± 0.28% vs 3.20 ± 1.02%) at 7 dpi and 5 dpi, respectively (Figures 8C,D), while CD4^+^CD8^+^ T and CD8 T cells showed impaired polyfunctionality for producing both TNF-α and IFN-γ (2.31 ± 1.0% vs 8.66 ± 1.92% and 6.57 ± 3.04% vs 12.39 ± 3.52%) at 7 dpi and 5 dpi, respectively (Figures 8A,B,E) in infected pigs in comparison with those of uninfected pigs. These findings demonstrated that αβ T cells from the ASFV-infected pigs were less responsive to stimulation and may be functionally impaired in vivo. However, there were still increased frequencies of TNF-α^+^IFN-γ^+^ and TNF-α^+^IL-2^+^ double-positive γδ T cells in infected pigs (Supplementary Figures S4A,B), though with no statistical significance due to huge individual differences. Further analysis of lymphocyte proliferation using Ki67 as a surrogate marker showed that B cells from the infected pigs significantly proliferated only at 3 dpi and then decreased (Supplementary Figure S5A) whereas CD4 T cells were less proliferative from 5 dpi on, compared to the control (Supplementary Figure S5B). The proliferation of CD8^+^, CD4^+^CD8^+^ T cells, γδ T cells and NK cells seemed not to be affected by ASFV infection (Supplementary Figures S5C–F).

### Regulatory T cells were reduced in periphery blood after ASFV infection

Regulatory T cell (Treg) is believed to be involved in the immunopathogenesis of ASF and affects the long-term protection of an attenuated ASFV vaccine (Kaser et al., 2011; Sanchez-Cordon et al., 2020). We also examined the frequency changes of CD3^+^CD4^+^Foxp3^+^ Tregs in periphery blood after ASFV CADC_HN09 infection. We found that the frequency of Tregs progressively decreased in the infected pigs and was significantly lower than that in the control group at 5 dpi (Supplementary Figures S6A,B), which is concomitant with the decreased proliferation of Treg (Ki67^+^ Treg) (Supplementary Figure S6C).

## Discussion

Virulent ASFV infection causes rapid death of the infected domestic pigs within 2 weeks (Zhao et al., 2019). Although the pathological changes of ASF have been extensively studied (Salguero et al., 2004; Wang et al., 2020; Li D. et al., 2021; Walczak et al., 2021), its immunopathogenesis remains incompletely understood. In addition to the cytokine storm that is believed to contribute to rapid death, *in vivo* death and activation of distinct immune cells have not been well tracked; moreover, it was unclear whether there was T-cell activation. In this study, we demonstrated that distinct immune cells underwent different levels of apoptosis kinetically *in vivo* over the course of infection and confirmed the cell types (B cells, CD4 T cells, monocytes, and dendritic cells) that were mainly affected by ASFV infection and accounted for the leukopenia documented in many studies (Carrasco de Lara et al., 1996a; Oura et al., 1998; Karalyan et al., 2012; Walczak et al., 2021). Interestingly, we found that T cells were transiently activated but eventually became less responsive to mitogenic stimulation, suggesting that the adaptive cellular immunity to ASFV might be initiated in the specific pathogen-unexperienced and pathogen-unimmunized piglets but somehow interrupted eventually. These findings improved our understanding of the immunopathogenesis of ASF.

The cellular immune response to the virulent ASFV strain has been examined in previous studies (Huhr et al., 2020; Schafer et al., 2021). CD4 T cells and CD79a^+^ B cells were shown to account for the lymphopenia, while CD4^+^CD8^+^ T cells were increased at 7 dpi in domestic pigs, and no T-cell activation was detected in terms of Ki67 and T-bet expression (Huhr et al., 2020). Consistent with this study, we also observed that B cells and CD4 T cells were the major populations that contributed to lymphopenia, but why and how ASFV induces B cell and CD4 T cell reduction or death require further investigation. In addition, different from the previous study, we demonstrated that there was T-cell activation during virulent ASFV infection, based on CD69 expression and *ex vivo* IFN-γ expression by T cells. However, this early activation of T cells seemed not to contribute to the protection as the infected pigs eventually died. One of the reasons for this could be that the initiation of anti-ASFV T cell immunity was interrupted by the apoptosis and reductions of antigen-presenting cells (Figures 1A,B, 2A,C) or overridden by ASFV-induced immunosuppression. Cytotoxic CD8 T cells are of great importance in antiviral infection. We failed to examine if there was an increase of perforin^+^ or granzyme^+^ effector CD8 T cell in acute infection, which was described elsewhere (Lagumdzic et al., 2023), due to a lack of antibodies.

It was well reported that ASFV induced immunosuppression (Dixon et al., 2019). ASFV-encoded proteins, such as pE199L, p54, and A179L, have been shown to induce cell death via apoptosis or necroptosis in cell lines (Hernaez et al., 2004; Li T. et al., 2021; Shi et al., 2021). TNF-α from ASFV-infected macrophages caused the apoptosis of bystander lymphocytes during later stages (Dixon et al., 2019). Whether the reduction of distinct immune cell subsets is directly mediated *in vivo* by viral proteins or induced by inflammatory cytokines still needs to be answered. Cytokine IL-1β and IL-18 are typically products of cell pyroptosis (Church, 2014; Schneider et al., 2017). In this study, we found that the concentration of IL-1β and IL-18 in the sera of the infected pigs was significantly increased, implying that pyroptosis may also be induced during ASFV infection. However, how a single viral protein induces the cell death of immune cells *in vivo* remains to be demonstrated.

Treg is generally induced during acute infection and plays a role in counteracting immunopathological lesions caused by the excessive inflammatory response (Kaser et al., 2011). In this study, we did not observe the same increase of Treg in periphery blood as in previous studies (Huhr et al., 2020; Schafer et al., 2021), though strong cytokine storm and neutrophil increases were documented (Wang et al., 2020; Franzoni et al., 2023). It is reasonable to speculate that lacking Treg to dampen aberrant inflammatory response may promote or accelerate the death of the infected pigs. Of note, individual variation among pigs and operational variations including blood collection, PBMC isolation, and disease progression between each time-point, which severely affected data consistency and interpretations, were minimized in this study by setting the uninfected pigs as a control group, something that was missing in previous studies (Huhr et al., 2020; Schafer et al., 2021).

It is debatable whether there is activation of adaptive immunity after virulent ASFV infection as the ASF disease progresses so rapidly that the infected pigs die quickly (Zhao et al., 2019). In this study, using two methods, CD69 expression and *ex vivo* cytokine staining, we showed that early activation of lymphocytes indeed had taken place after ASFV CADC_HN09 infection. However, the capability of T cells to produce cytokines was impaired later. Apart from the cell death of activated T cells, the impairment of antigen presentation could be another possible reason. It was reported that ASFV infection did not significantly change the expression level of MHC II on mature pig bone marrow cells (pBM) and Mφ (Lithgow et al., 2014; Franzoni et al., 2017). However, we found that the expression of MHC II on monocyte and cDC1 was significantly decreased after ASFV infection (Figure 4), suggesting that antigen presentation by these cells may be impaired, thus disrupting the elicitation of effective adaptive immunity to ASFV. In addition, B cell reduction and CD21 downregulation on B cells may account for the failure of developing an effective humoral response as CD21 is involved in the regulation of B cell activation (Tedder et al., 1994).

Overall, in the present study, by examining the cellular changes of domestic pigs after infection with the highly virulent ASFV CADC_HN09 strain, we found that ASFV infection induced different levels of apoptosis of distinct immune cells over the course of infection. There was early T-cell activation, but T-cell function was impaired later. The failure to develop an effective adaptive immunity to ASFV may be related to ASFV-induced immunosuppression or the impaired function of APCs, B cells, and T cells, leading to a dual impairment of humoral and cellular immune response. This study provides new insights into the rapid disease progression of ASF in domestic pigs, improves our understanding of the immunopathogenesis of ASF, and paves the way for further investigation.

## Data availability statement

The original contributions presented in the study are included in the article/Supplementary material, further inquiries can be directed to the corresponding authors.

## Ethics statement

The animal study was approved by the Animal Welfare and Ethics of China Animal Disease Control Center. The study was conducted in accordance with the local legislation and institutional requirements.

## Author contributions

YT: Data curation, Formal analysis, Investigation, Methodology, Software, Validation, Visualization, Writing – original draft. DW: Data curation, Investigation, Validation, Writing – original draft. SH: Resources, Writing – original draft. ZC: Investigation, Writing – original draft. WL: Data curation, Writing – original draft. FJ: Data curation, Writing – original draft. YS: Data curation, Writing – original draft. YH: Data curation, Writing – original draft. XW: Investigation, Writing – original draft. QW: Investigation, Writing – original draft. SQ: Investigation, Writing – original draft. JWa: Data curation, Writing – original draft. TL: Data curation, Writing – original draft. XH: Methodology, Writing – original draft. JZ: Resources, Writing – original draft. JWu: Funding acquisition, Project administration, Supervision, Writing – review & editing. SS: Project administration, Supervision, Conceptualization, Funding acquisition, Writing – review & editing. XZ: Project administration, Supervision, Writing – original draft.
