# Supplementary material for: Immune cell early activation, apoptotic kinetic, and T-cell functional impairment in domestic pigs after ASFV CADC_HN09 strain infection
Source: Front Microbiol. 2024 Feb 14;15:1328177. doi: 10.3389/fmicb.2024.1328177 (PMC10899498; doi:10.3389/fmicb.2024.1328177)
Supplement: Supplementary file 1 [file Data_Sheet_1.docx]

Supplementary Material

## Supplementary Figures


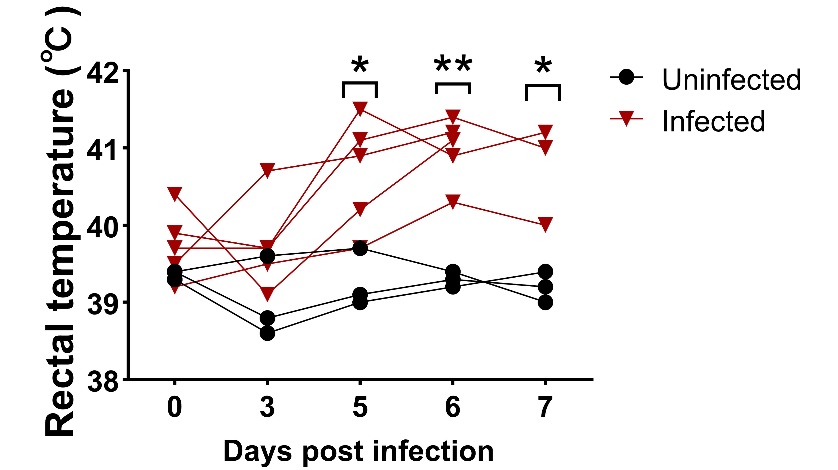


**Supplementary Figure 1** ASFV infection caused increased body temperature in piglets. Eight 2-month-old piglets were randomly divided into two groups (one uninfected group of 3 and one infected group of 5). Rectal temperature of all pigs was daily collected before and post infection. Each point represents data from a single pig. Statistically significant difference is shown, *P <0.05. **P <0.01.


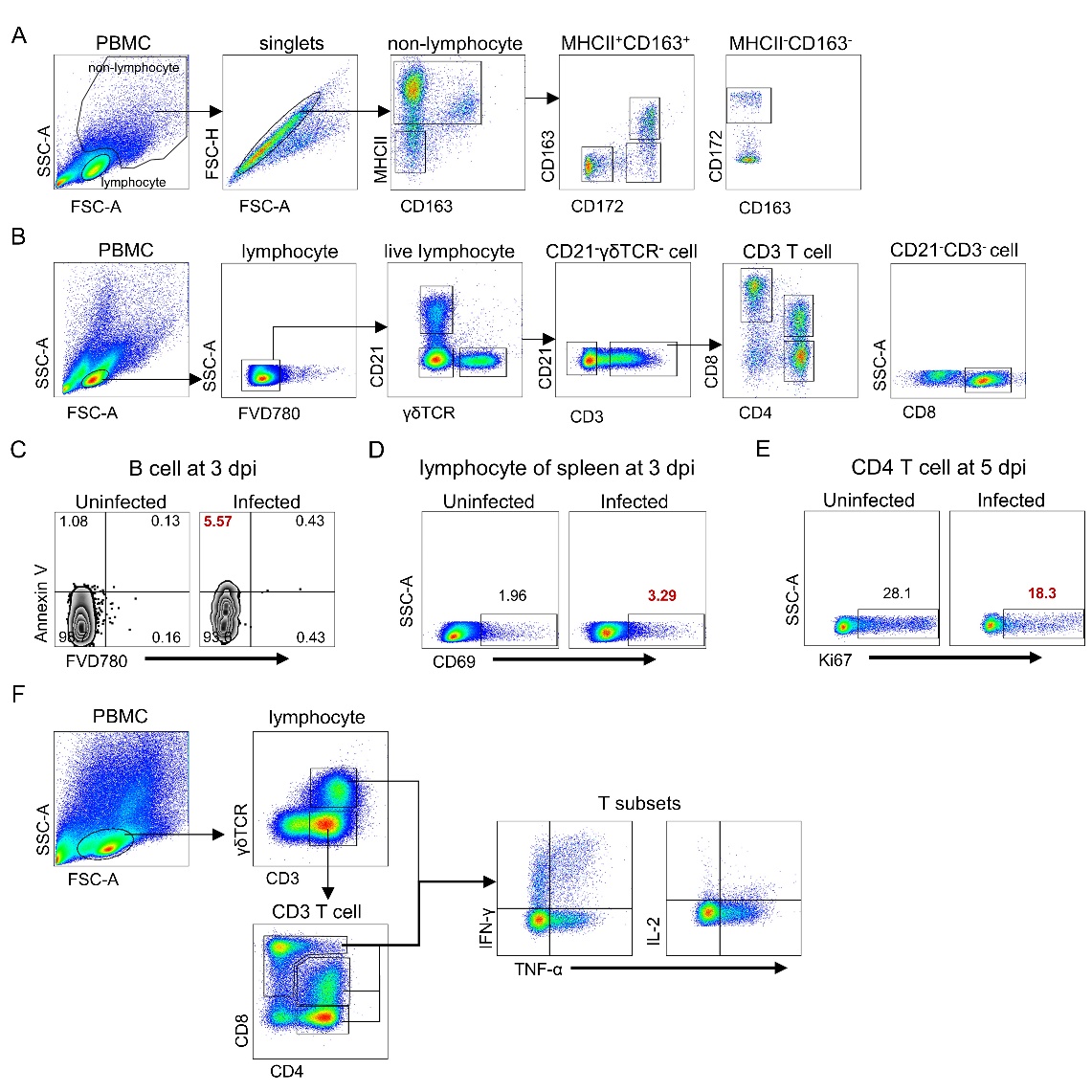


**Supplementary Figure 2** Gating strategy of monocyte, DC subsets, neutrophil and lymphocyte subsets in FCM analysis. (A) Monocyte (MHCII^+^CD163^+^CD172a^+^), cDC1(MHCII^+^CD163^-^CD172a^-^) and cDC2 (MHCII^+^CD163^-^CD172a^+^) are gated on single PBMC excluded lymphocyte counterpart (non-lymphocyte). Neutrophil (MHCII^-^CD163^-^CD172^+^) are gated on MHCII^-^CD163^-^ non-lymphocyte. (B) CD21^+^ B cell and γδ T cell (γδTCR^+^) are gated on FVD780^-^ lymphocyte (live lymphocyte). T cell subsets (CD4 T, CD8 T, CD4^+^CD8^+^ T cell) are gated on CD21^-^γδTCR^-^CD3^+^ T lymphocyte. NK cells (CD21^-^CD3^-^CD8α^+^) are gated on CD21^-^γδTCR^-^CD3^-^ lymphocyte. FVD780^+^ cells, dead cells. (C) Representative zebra plots of B cell apoptosis at 3 dpi, Annexin V^+^ cells, apoptosis cells, while FVD780^+^cells mean dead cells. Representative pseudocolor plots of CD69^+^ lymphocyte in spleen at 3 dpi (D) and plots of the proliferating CD4 T cell at 5 dpi (E). (F) Gating strategy for cytokine expression analysis. IFN-γ^+^-, IFN-γ^+^TNF-α^+^-, TNF-α^+^-, TNF-α^+^IL-2^+^- and IL-2^+^ γδ T cell, CD4 T, CD4^+^CD8^+^ T and CD8 T cell are gated on lymphocyte and γδTCR^-^CD3^+^ T cells, respectively.


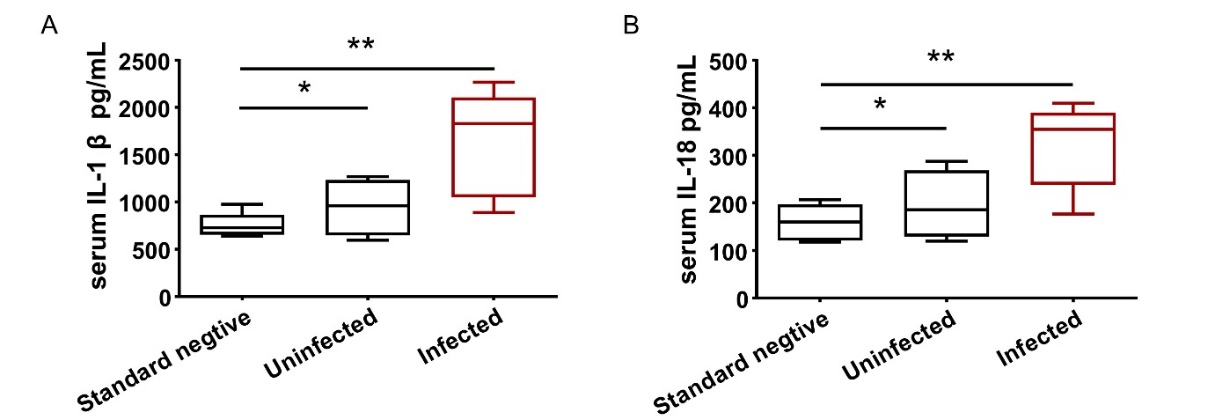


**Supplementary Figure 3** Increased levels of IL-1β and IL-18 in pig serum infected with ASFV. Pig serum was collected at 5 dpi and was used for determination of IL-1β and IL-18 by ELISA. (A) Porcine IL-1β level in serum at 5 dpi. (B) Porcine IL-18 level in serum at 5 dpi. Data shown are mean ± SD. *P <0.05. **P <0.01.

**
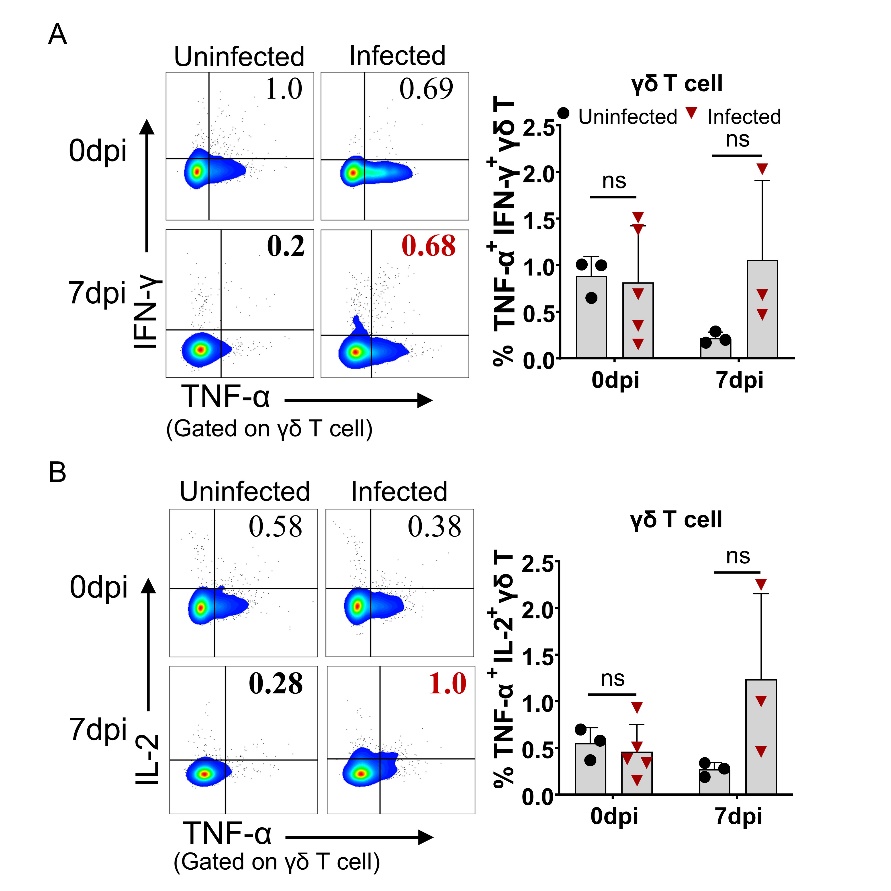
**

**Supplementary Figure 4** ASFV infection affected the capability of γδ T cell to produce cytokines. At the indicated time points, PBMCs of ASFV-infected and uninfected piglets were separated and stimulated with mitogen and were stained for lymphocyte subsets, followed by an intracellular cytokine staining step and cells were determined by FCM. Representative pseudocolor plots (left panels) and the differential frequencies of TNF-α^+^IFN-γ^+^ γδ T cells (A) and TNF-α^+^IL-2^+^ γδ T cells (B) (right panels). Each point represents data from a single pig while bars represent the mean of each group. Data shown are mean ± SD. ns, no statistical significance.


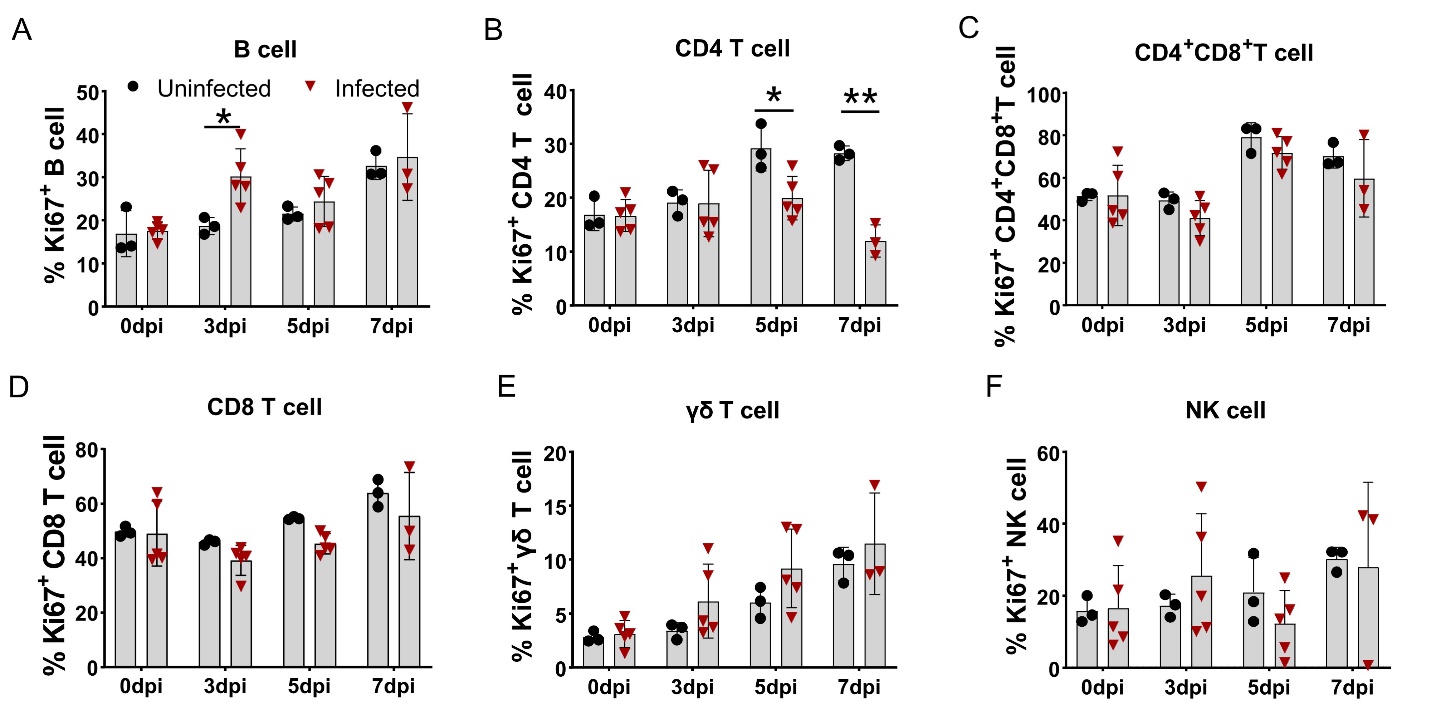


**Supplementary Figure 5** ASFV infection had different effects on lymphocyte proliferation. At the indicated time points, PBMC was prepared from blood of uninfected and infected pigs and was stained for lymphocyte subsets, followed by a staining step of intranuclear Ki67 and analyzed by FCM. The percentages of Ki67^+^- B cell (A), CD4 T cell (B), CD4^+^CD8^+^ T cell (C), CD8 T cell (D), γδ T cell (E) and NK cell (F). Each point represents data from a single pig while bars represent the means for the designated time points. Data shown are mean ± SD. *, p < 0.05. ***, p < 0.001.


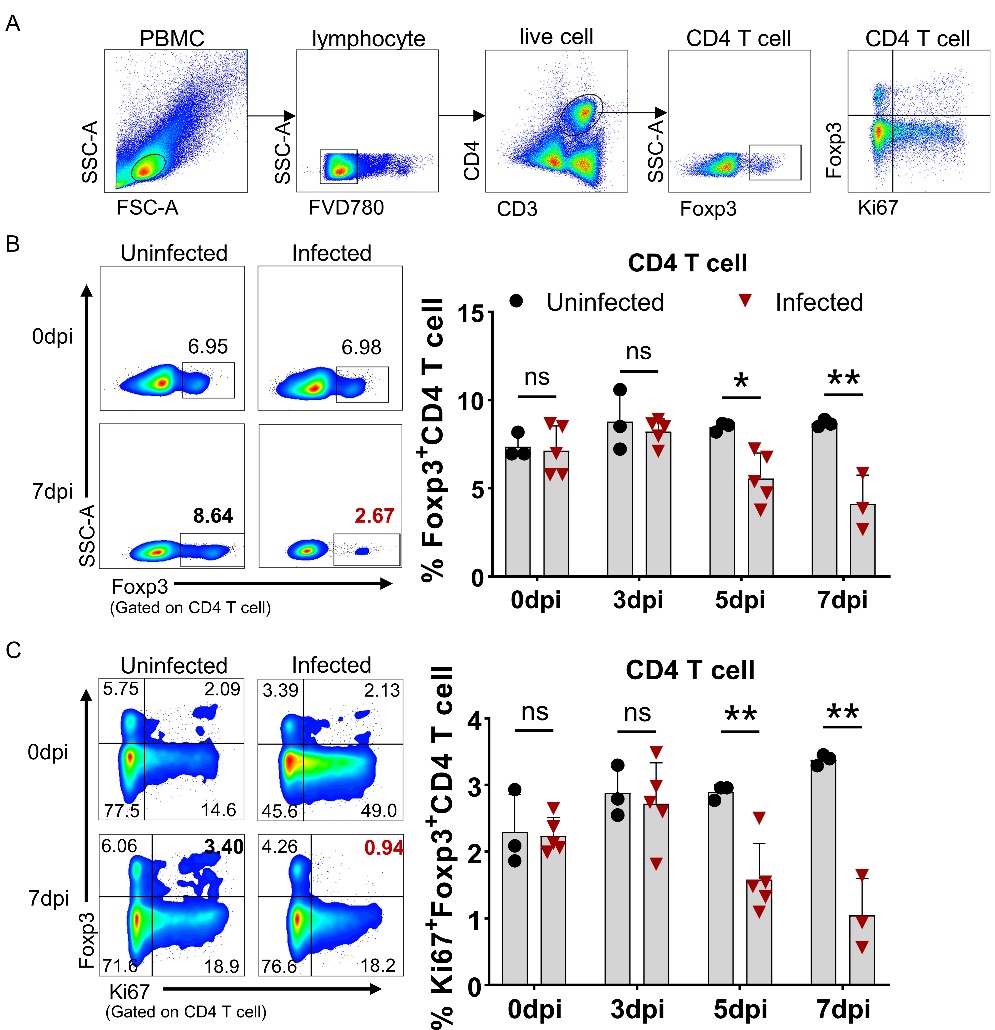


**Supplementary Figure 6** The frequency of Treg decreased after ASFV infection. At the indicated time points, PBMCs were prepared from blood of pigs and were stained for lymphocyte subsets and intranuclear Foxp3 and Ki67 and were analyzed by FCM. (A) Gating strategy for Treg. Treg was subdivided based on its expression of Foxp3 and CD4 markers on CD3 T cell and was investigated for expression of Ki67. FVD780^+^ cells, dead cells. (B) Representative pseudocolor plots (left panel) and frequencies of Treg (Foxp3^+^CD4 T cell) in CD4 T cell (right panel). (C) Representative pseudocolor plots (left panel) and frequencies of proliferating Treg (Ki67^+^Foxp3^+^CD4 T cell) in CD4 T cell (right panel). Data shown are mean ± SD. *, p < 0.05. **, p < 0.01. ***, p < 0.001.
